# A comparative study of cancer proteins in the human protein-protein interaction network

**DOI:** 10.1186/1471-2164-11-S3-S5

**Published:** 2010-12-01

**Authors:** Jingchun Sun, Zhongming Zhao

**Affiliations:** 1 Department of Biomedical Informatics, Vanderbilt University School of Medicine, Nashville, TN 37232, USA; 2 Bioinformatics Resource Center, Vanderbilt-Ingram Cancer Center, Vanderbilt University, Nashville, TN 37203, USA; 3 Department of Cancer Biology, Vanderbilt-Ingram Cancer Center, Vanderbilt University, Nashville, TN 37232, USA

## Abstract

**Background:**

Cancer is a complex disease. So far, many genes have been reported to involve in the development of cancer. Rather than the traditional approach to studying individual genes or loci, a systematic investigation of cancer proteins in the human protein-protein interaction network may provide important biological information for uncovering the molecular mechanisms of cancer and, potentially, other complex diseases.

**Results:**

We explored global and local network characteristics of the proteins encoded by cancer genes (cancer proteins) in the human interactome. We found that the network topology of the cancer proteins was much different from that of the proteins encoded by essential genes (essential proteins) or control genes (control proteins). Relative to the essential proteins or control proteins, cancer proteins tended to have higher degree, higher betweenness, shorter shortest-path distance, and weaker clustering coefficient in the human interactome. We further separated the cancer proteins into two groups (recessive and dominant cancer proteins) and compared their topological features. Recessive cancer proteins had higher betweenness than dominant cancer proteins, while their degree distribution and characteristic shortest path distance were also significantly different. Finally, we found that cancer proteins were not randomly distributed in the human interactome and they connected strongly with each other.

**Conclusion:**

Our study revealed much stronger protein-protein interaction characteristics of cancer proteins relative to the essential proteins or control proteins in the whole human interactome. We also found stronger network characteristics of recessive than dominant cancer proteins. The results are helpful for cancer candidate gene prioritization and verification, biomarker discovery, and, ultimately, understanding the etiology of cancer at the systems biological level.

## Background

Cancer is a common complex disease. Many genetic factors and genes have been reported to play an important role in its pathogenesis. Identification of genes that activate or accelerate the development of cancer (referred as ‘cancer genes’) has been one of the major goals in cancer research. During the past three decades, a great number of cancer genes have been reported. Recently, a census of human cancer genes was conducted based on the published literature [1] and the annotated genes were stored in the Cancer Gene Census database [2]. Although this collection of genes is still likely incomplete or error-prone, it offers us a great opportunity to examine the general features of many cancer genes, rather than individual gene, at one time. Indeed, several important features have been found based on these genes, such as sequence alteration, mutations in different cancers, protein domains and partial network properties [1,3].

Recently, investigation of interactions between proteins encoded by disease genes in the human protein-protein interaction network (i.e., the human interactome) has become one of the major and powerful approaches to elucidating the molecular mechanisms underlying the complex diseases [4-10]. Cancer is the most severe human disease, costing us many billions of dollars each year. Thus, a systematic examination of the proteins encoded by cancer genes (cancer proteins) in the human interactome may help us identify new candidate genes, improve candidate gene prioritization methods, and have a deeper understanding of the genetic landscape of cancer. This research topic emerged recently. For example, we have seen recent reports on the exploration of cancer related networks including the topological analysis [3,11], prediction of cancer candidate genes [12] and their dynamic modularity [13]. Meanwhile, several studies reported that essential genes tended to encode hubs [14,15], but another study revealed that vast majority of disease genes are nonessential and have no tendency toward higher degree in the human protein-protein interaction network. Such incongruence of disease and essential gene properties in the human interactome requires further investigations. Moreover, so far these studies have explored only limited network properties or focused on one specific type of cancers. To have a global view of the interactions of cancer proteins, it is necessary to systematically investigate the network properties of cancer proteins in the human interactome, to compare properties of the subtypes of cancer proteins, and also to compare cancer proteins with essential proteins.

In this study, we explored the global and local network characteristics of cancer proteins through mapping the cancer proteins into the human interactome. For the global properties, we performed four different topological measurements, i.e., degree, betweenness, clustering coefficient and shortest-path distance. To assess the observed topological features of cancer proteins, we compared them to other proteins encoded by essential genes (essential proteins) or control genes (control proteins). For local network characteristics, we extracted subnetworks for the cancer proteins and compared them with the randomly generated networks. Our results revealed that (1) cancer proteins display a global topology that is significantly different from that of essential proteins or control proteins, (2) cancer proteins could form non-random networks, and (3) recessive cancer proteins had even stronger network characteristics than dominant cancer proteins.

## Methods

### Construction of the human interactome

Investigation of the protein interaction characteristics for a set of proteins requires a complete and accurate whole protein interaction network. In this study, we integrated all the available human protein-protein interactions from six major protein interaction databases but restricted only those with experimental evidence. The six databases are Human Protein Reference Database (HPRD, release 7) [16], Biomolecular Interaction Network Database (BIND, 20060525) [17] , IntAct (downloaded on 2007-09-28) [18], Molecular INTeration database (MINT, downloaded on 2007-06-28) [19], Reactome (version 24) [20] and the Database of Interacting Proteins (DIP, version Hsapi20070707) [21]. After removing the redundancy and self-interactions, we constructed a comprehensive and reliable human protein-protein interaction network, which contained 53,255 unique interactions among 10,549 proteins. We considered these interactions as an approximation of a full set of human protein-protein interactions, or, the human interactome.

### Cancer genes, essential genes and control genes

We obtained 384 cancer genes and their detailed annotations from the Cancer Gene Census database (2009-08-04 version) [2]. The Cancer Gene Census database is updated regularly based on the original census data summarized in Nature Review Cancer [1]. Among the 384 cancer genes, 382 could be mapped to genes with official gene symbols in the National Center for Biotechnology Information (NCBI) [22]. We considered these genes as cancer candidate genes. Among them, there were 342 genes whose proteins could be found in the human interactome. A cancer gene may act in a dominant or recessive manner [23]. Therefore, we separated these 342 cancer genes into two groups: recessive group, which had 69 cancer genes, and dominant group, which had 273 cancer genes, according to the annotations in the Cancer Gene Census database.

For comparison, we compiled two other gene sets: essential genes and control genes. For essential gene set, we used the method described in Goh et al. [7], which considered the classes of embryonic/prenatal lethality and postnatal lethality as lethal phenotypes and the rest of phenotypes as non-lethal ones. We retrieved the human-mouse orthologs and mouse phenotype data from the Mouse Genome Informatics (downloaded on September 5, 2008) [24,25]. There were 2217 mouse-lethal human orthologs. We considered them as essential genes. Among them, 1896 were mapped in the human interactome. For control genes, we excluded the cancer genes and essential genes from all protein-coding genes mapped in our human interactome. This resulted in 8402 control genes. Table 1 summarizes the five gene sets and their mapped information in the human interactome.

**Table 1:** Summary of network properties for five protein sets in the human interactome

Protein set	No. of genes	No. of proteins^a^	Network properties (average value)				

			Connectivity	Betweenness (10^4^)	Clustering coefficient	gSPD^b^	cSPD^c^
Cancer proteins	382	342	25.41	6.29	0.13	3.63	3.20
Essential proteins	2217	1896	18.39	4.16	0.14	3.76	3.55
Control proteins	8402	8402	8.25	1.47	0.19	3.99	4.10
Recessive cancer proteins	72	69	33.03	9.82	0.13	3.61	3.05
Dominant cancer proteins	310	273	23.44	5.33	0.13	3.64	3.21

^a^Number of genes whose proteins could be found in the human interactome.

^b^General shortest-path distance between the interest proteins and the remaining proteins in the whole network.

^c^Characteristic shortest-path distance among the interest proteins in the whole network.

### Global network analysis

For each node in each protein set, we applied four topological measures to assess its role in the network [26]: degree and degree distribution, clustering coefficient, betweenness, and shortest-path distance. First, the degree or connectivity of a node, *k*, is the count (number) of the direct links of this node in the network. For a set of proteins, we plotted degree distribution for more detailed information. Based on the distribution, we had *P(k*), which is the probability that a node has exactly *k* links in the set of proteins. The more links a node has, the more important it is in terms of network stability [27]. Second, the clustering coefficient, *C,* of a node is the ratio of the observed number of direct connections between the node’s immediate network neighbors over the maximum possible number of such connections. Third, the betweenness of a node, *B*, is defined as the number of shortest paths between all possible pairs of nodes in the network that traverse the node. In a biological network, betweenness measures the ways in which signals can pass through the interaction network [13]. Fourth, for a pair of selected nodes in the network, there are many alternative paths between them. The path with the smallest number of links is defined as the shortest path. The number of links passing through in the shortest path is defined as shortest-path distance (*L*). Because the two nodes in a pair might belong to the same protein set or to different protein sets, we extended the shortest paths into two categories. The first one is the shortest-path between one node belonging to one protein set and another node not belonging to that protein set. The distance was defined as the general shortest-path distance (abbreviated as gSPD). The second one is the shortest-path between two nodes from the same protein set. The distance was defined as the characteristic shortest-path distance (abbreviated as cSPD). For a protein set, we calculated the mean value of all proteins, i.e., average degree, average betweenness, average clustering coefficient or average shortest-path distance.

### Construction and randomization of cancer-specific networks

To generate cancer-specific network, we started from the 342 cancer proteins and extracted the protein-protein interactions between these proteins. To test whether the observed cancer-specific networks are not random, we applied an empirical approach. First, we generated 1000 randomized networks with the same number of nodes and links as in the cancer-specific networks using the Erdos-Renyi model [28] in R igraph package. For each random network, we calculated the average values for three measurements: betweenness (*B*), clustering coefficient (*C*), and general shortest-path distance (*L*). Next, we counted the number of the randomized networks whose average betweenness (*n_B_*) or clustering coefficient (*n_C_*) was higher than the observed betweenness or clustering coefficient, respectively. For general shortest-path distance, we counted the number (*n_L_*) of the randomized networks whose shortest-path distance is shorter than that of the cancer-specific networks. Finally, we calculated empirical *P* value = *n_B_* /1000, *n_C_*/1000, or *n_L_* /1000, respectively, for these three network topological measurements.

### Functional analysis of top cancer proteins

We examined the functional features of cancer proteins that were top ranked in network analysis. We ranked the cancer proteins and selected the top 20 cancer proteins for each network measure. This resulted in a total of 44 cancer proteins after removing the redundancy. We examined the biological significance of these top genes in KEGG database by pathway enrichment analysis. KEGG is a database of biological system that integrates genomic, chemical and functional information through the process of PATHWAY mapping [29]. KEGG PATHWAY database includes about 120 existing pathways and KEGG DISEASE links disease genes, pathways, drugs and diagnostic markers [30]. We calculated *P* values using the Fisher’s exact test using the human genome as the background and then adjusted the *P* values by FDR using Benjamini- Hochberg procedure [31]. We applied the following two criteria to identify cancer-enriched pathways: 1) FDR *P* value was less than 0.001 and 2) the number of cancer proteins involved in a pathway was at least 5.

## Results and discussion

### Global properties of cancer proteins

In a biological network, topological properties of nodes are important for understanding the underlying biological mechanisms [26]. In this study, we examined four important network measurements: degree, betweenness, clustering coefficient and shortest-path distance. Table 1 summarizes the global properties of the five protein sets.

#### Cancer proteins tend to have higher degree

The average degree of the 342 cancer proteins was 25.41, which was significantly higher than that of the essential proteins (18.39, Wilcoxon test, *P* = 1.2 × 10^–6^) or that of the control proteins (8.25, *P* < 2.2 × 10^–16^). The average degree of the cancer proteins is approximately 3.1 times that of the control proteins. The ratio is higher than that (2.1) based on the predicted protein-protein interactions in a previous study [3]. Thus, proteins encoded by cancer genes tend to interact strongly with other proteins and have higher connectivity in the whole network. This observation of higher degree in the cancer proteins than the control proteins supports a recent report that disease genes tend to have higher degree than non-disease genes [3].

**Figure 1:**
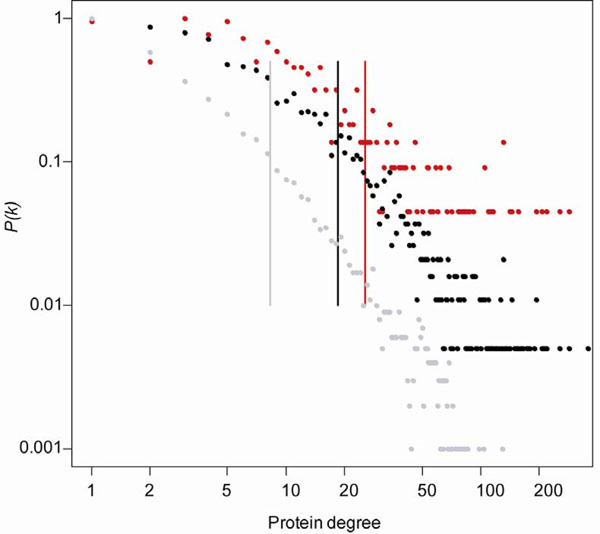
**Degree distribution and average degrees of the cancer, essential, and control proteins.** Y-axis represents the proportion of proteins having a specific degree. The average degree of each protein set is labelled in vertical line. Red dots denote cancer proteins, black dots denote essential proteins, and grey dots denote control proteins.

To have a more detailed view of the degree characteristics, we plotted the degree distribution for the three proteins sets (Figure 1). The cancer proteins tended to skew toward higher degree than the essential proteins or control proteins (cancer vs. essential proteins, *P* = 1.1 × 10^–11^; cancer vs. control proteins, *P* < 2.2 × 10^–16^, Wilcoxon test). Highly connected nodes are usually defined as “hubs”. At present, definition of hubs is still an unsolved issue in biological network analysis. We applied two cutoffs (degree >5 and degree >12) to define hubs in this study. The first cutoff ( degree >5) is the traditional definition of hubs in the protein interaction network [13]. According to this cutoff, there were 250 (73.1%) of the cancer proteins classified into hubs, which is significantly higher than that of the essential proteins (61.4%, χ^2^ test, *P* = 4.7 × 10^–5^) or that of the control proteins (35.4%, *P* = 3.3 × 10^–45^). The second cutoff (degree >12) was determined by the method in Yu et al. [15], which selects the cutoff based on degree distribution. According to this cutoff, there were 164 cancer proteins (48.0%) being classified into hubs. The proportion was statistically higher than that of the essential proteins (38.1%, χ^2^ test, *P* = 7.3 × 10^–4^) or the control proteins (16.6%, *P* = 2.1 × 10^–49^). On average, a protein with high degree is approximately two times more likely to be involved in cancer than in control. These observations indicated that the cancer proteins were more likely to be network hubs than the control proteins or even the essential proteins.

Interestingly, comparing the essential proteins, the cancer proteins had stronger connectivity and tended to be hubs in the human interactome. This observation might reflect that the cancer genes mainly play important role in cell proliferation, cell differentiation and cell death [1], or might be due to the data bias of the cancer genes toward more studies, as cancer is the most studied disease [4]. Our preliminary study using the homologous genes in yeast and unbiased yeast protein-protein interaction data revealed that this data bias is not the primary factor for the cancer protein features and the conclusion above could still hold in the yeast interactome (data not shown). More work is needed to elucidate this difference.

#### Cancer proteins tend to have higher betweenness

**Figure 2:**
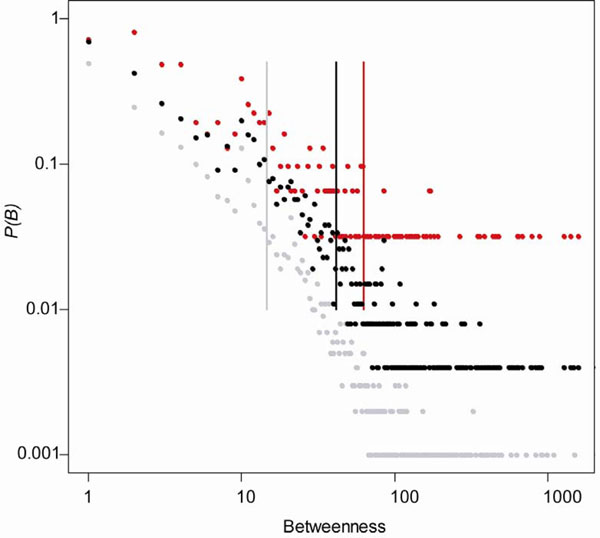
**Betweenness distribution of the cancer, essential, and control proteins.** Y-axis represents the proportion of proteins having a specific betweenness. The average betweenness value of each protein set is labelled in vertical line. Red dots denote cancer proteins, black dots denote essential proteins, and grey dots denote control proteins.

Betweenness measures the number of shortest paths through a node in a network. This measurement may reflect the extent of signals that might have paths through the node in a biological network. Among the 342 cancer proteins, 319 had betweenness greater than zero. The average betweenness of the cancer proteins (6.29 × 10^4^) was significantly greater than that of the essential proteins (4.16 × 10^4^, Wilcoxon test, *P* = 1.9 × 10^–4^) or that of the control proteins (1.47 × 10^4^, *P* < 2.2 × 10^–16^). Figure 2 shows the betweenness distributions of the three protein sets. The pattern of betweenness distributions was similar to that of the degree distributions for the three protein sets. The cancer proteins tended to have higher betweenness compared to the essential proteins or control proteins, reflecting that there are more signals passing through the cancer proteins compared to the essential proteins or control proteins.

#### Cancer proteins tend to have lower clustering coefficient

**Figure 3:**
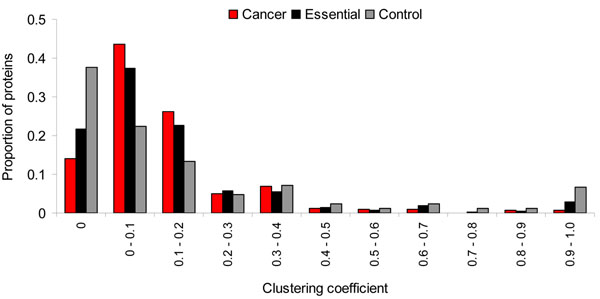
Distribution of clustering coefficients of the cancer, essential, and control proteins.

Clustering coefficient of a node of interest in a network measures how well connected among its direct interactors. A higher clustering coefficient of a node indicates a higher density of its network connections. The average clustering coefficient of the cancer proteins (0.13) was almost equal to that of the essential proteins (0.14, Wilcoxon test, *P* = 0.20) but was significantly lower than that of the control proteins (0.19, *P* = 0.0024). To explore specific features of clustering coefficients, we separated clustering coefficients into different bins with an interval of 0.1 and calculated the relative frequency of proteins in each bin (Figure 3). We found that 69.8% of the cancer proteins had their clustering coefficients within the intervals (0-0.2] (excluding 0 but including 0.2). This compared to 35.8% of the control proteins or 60.0% of the essential proteins. Interestingly, when the clustering coefficient was 0 or greater than 0.9, the control proteins had highest proportion but cancer proteins had the lowest proportion among the three protein sets (Figure 3). Overall, relative to the control proteins, the neighbors of the cancer proteins had less likelihood to connect each other.

#### Cancer proteins tend to have shorter shortest-path distance

**Figure 4:**
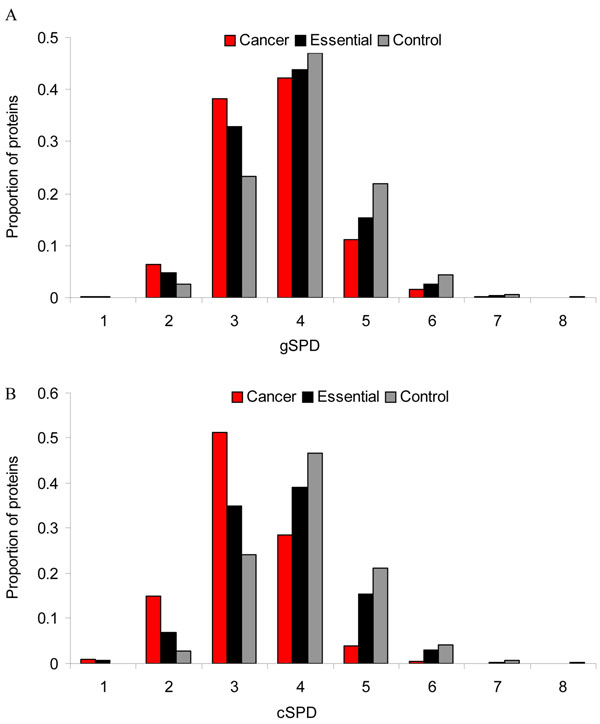
**Distribution of the shortest-path distance of the cancer, essential, and control proteins. (A)** General shortest-path distance (gSPD) between nodes of interest and the rest of nodes in the whole network. **(B)** Characteristic shortest-path distance (cSPD) between a pair of nodes from the same protein set.

The average general shortest-path distance (gSPD) of the cancer proteins was 3.63, which was significantly shorter than that of the essential proteins (3.76, Wilcoxon test, *P* = 1.1 × 10^–6^) or the control proteins (3.99, *P* < 2.2 × 10^–16^). Figure 4A shows the distribution of the general shortest- path distance for the cancer proteins, essential proteins and control proteins. When the distance was ≤ 3, the proportion of the cancer proteins (44.9%) was larger than that of the essential proteins (37.9%) or that of the control proteins (25.9%). The opposite pattern was observed when the distance was >3. Therefore, the overall shorter average general shortest-path distance for cancer proteins is primarily attributed to those cancer proteins whose gSPD distance is ≤ 3 in the comparison of the essential or control proteins.

Similarly, we found that the average characteristic shortest-path distance (cSPD) of the cancer proteins (3.20) was significantly shorter than that of the essential proteins (3.55, Wilcoxon test, *P* < 2.2 × 10^–16^) or that of the control proteins (4.10, *P* < 2.2 × 10^–16^) (Figure 4B). For the cancer proteins, the average cSPD (3.20) was significantly shorter than the average gSPDs (3.63) (P < 2.2 × 10^–16^). Specifically, when distance equaled to 3, the proportion of the cancer proteins for gSPD (38.3%) was much lower than that of the cancer proteins for cSPD (51.3%). Conversely, when distance equaled to 4, the proportion of the cancer proteins for gSPD (42.2%) was much larger than that of the cancer proteins for cSPD (28.5%). This comparison indicates that the efficiency of cancer proteins contacting with each other might be higher than that of cancer proteins contacting with non-cancer proteins in the human protein interaction network.

### Strong positive correlation between degree and betweenness of cancer proteins

**Figure 5:**
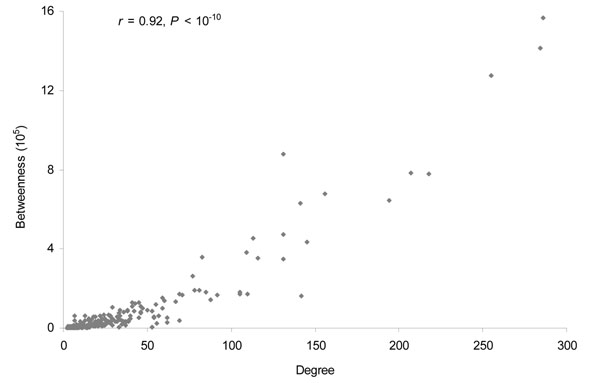
Significant correlation between degree and betweenness of the cancer proteins.

Since betweenness measures the paths through a node which has its degree of interactions in the network, it is interesting to examine the correlation between degree and betweenness for cancer proteins. Figure 5 plotted the degree and betweenness for all cancer proteins. We found the correlation between the degree and betweenness was very strong (Pearson’s product-moment correlation test, r = 0.92, *P* < 2.2 × 10^–16^). The result indicates that the number of the paths through a cancer protein in the human interactome is highly correlated with its degree.

### Weak negative correlation between degree and clustering coefficient of the cancer proteins

Clustering coefficient is based on degree [26]. Similarly, we examined the correlation between degree and clustering coefficient for cancer proteins. The correlation coefficient (r) was -0.14 (Pearson’s product-moment correlation test, *P* = 0.01) between these two measurements. This negative correlation confirms our previous observation that, although cancer proteins themselves have high connectivity, relative to other proteins and cancer proteins’ degree, their neighbors do not have high density of clusters (Table 1).

### Global properties of the recessive and dominant cancer proteins

Among the 72 recessive and 310 dominant cancer genes, 69 and 310 whose proteins could be mapped into the human interactome, respectively. We analyzed their global properties. The results were summarized in Table 1. For average degree, average clustering coefficient and general shortest-path distance (gSPD), no significant difference was detected between the recessive and dominant cancer proteins. The average degree of the recessive cancer proteins (33.03) was higher than that of recessive cancer proteins (23.44). Although the average degree was not significantly different between these two cancer protein sets (Wilcoxon test, *P* = 0.11), we found that their degree distributions were significantly different (Wilcoxon test, *P* = 3.1 × 10^–8^). Moreover, the average betweenness (9.82 × 10^4^) of the recessive cancer proteins was higher than that of the dominant cancer proteins (5.33 × 10^4^, Wilcoxon test, *P* = 0.049). Finally, the average cSPD of the recessive cancer proteins (3.05) was significantly lower than that of the dominant cancer proteins (3.21, Wilcoxon test, *P* < 2.2 × 10^–16^). These comparisons revealed that recessive cancer proteins have even stronger network topological properties, suggesting different inheritable mechanisms of the recessive and dominant cancer genes in cancer.

### Nonrandomness of cancer-specific subnetworks

**Figure 6:**
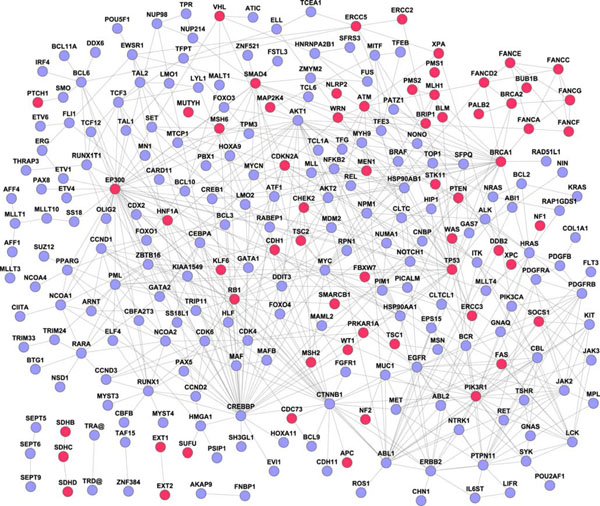
**Cancer-specific subnetworks extracted from the human interactome based on their direct interactions.** Recessive cancer proteins are labelled in red and dominant cancer proteins in blue. Two hundreds and forty cancer proteins could form a large network while the remaining 14 cancer proteins formed six small networks.

To gain further insight into the organization and environment of the cancer proteins, we extracted cancer specific subnetworks from the human interactome. These subnetworks included all protein-protein interactions between the cancer proteins. We did not include any other non- cancer proteins because the network would be otherwise too large. Among the 342 cancer proteins, 254 (74.3%) had direct links (a total of 595 direct links). Among these 254 cancer proteins, 240 could form a large subnetwork while the other 14 proteins formed 6 small subnetworks. Figure 6 displays these subnetworks with two different types of cancer proteins being marked: recessive cancer proteins in red and dominant cancer proteins in blue. In the cancer-specific networks, the average connectivity was 4.69, the average general shortest-path distance was 3.57 and the average clustering coefficient was 0.20.

We next assessed whether the cancer-specific subnetworks are non-random. We generated 1000 random subnetworks with the same number of nodes and links in the cancer-specific networks, and then compared their average degree, average shortest-path distance and average clustering coefficient. Among the 254 nodes in cancer specific subnetworks, 62 (22.4%) had high connectivity (degree > 5), while among the 254,000 nodes in the 1000 random subnetworks, only 0.2% of the nodes had high connectivity (degree > 5). Among 1000 random networks, the average shortest-path distance was 3.71 and the average clustering coefficient was 0.02. The empirical *P* values for shortest-path distance and clustering coefficient were zero. This evaluation revealed that the cancer-specific networks are not random.

It is worth noting that, in the cancer subnetworks, several cancer proteins are critical in the network topology: BRCA1, EP300, PIK3R1 and TP53. All of them are recessive cancer proteins. When we removed them from the cancer-specific network, the network centralization decreased from 0.141 to 0.126. Here, network centralization measures the degree of the effect when removing some central nodes in the whole network [32]. The result indicates that after removing the four proteins from the cancer-specific network, the cancer-specific network become much looser. This network feature supports the experimental evidence of these genes being critical in the pathology of cancer.

### Pathways enriched in top cancer proteins

Based on the comparison of the basic network properties among cancer proteins, essential proteins and control proteins, we selected the top 20 cancer proteins for each network measurement (connectivity, betweenness, clustering coefficient, gSPD and cSPD). This resulted in a total of 44 non-redundant cancer proteins. The strong overlap of top cancer proteins among these five network measurements suggests that these network properties are effective in detecting cancer protein features.

To examine functional features of these top cancer genes, we performed pathway enrichment analysis using KEGG data [29] (see Methods). We identified 24 pathways that were significantly enriched with the 44 top cancer genes (Fisher’s exact test, FDR *P* < 0.001). These pathways were listed in Table 2. Among these 24 pathways, 13 (54.2%) were directly related to cancer while the remaining ones were related to cell cycle/division, adhesion and signaling, which are also related to cancer. It is worthy noting that all these 13 pathways were ranked in the top 15 of the pathways ranked by FDR *P* values. Among the remaining 11 pathways, 7 were annotated to be part of cancer category in humans in KEGG PATHWAY database [30]. They were related to the biological processes such as sustained angiogenesis, evading apoptosis and proliferation, which are central processes of tumorigenesis [33]. This analysis indicates that the cancer genes populate cellular pathways that control cellular proliferation and cell death [34]. In summary, functional features of the top cancer genes support that pathology of cancer is a dynamic process caused by dysregulation of multiple pathways.

**Table 2:** Pathways significantly enriched in the top cancer proteins ranked by network properties

Ranking	KEGG entry	Pathway name	No. of gene^a^	*P* value^b^	FDR *P* value^c^
1	hsa05215	Prostate cancer	15	1.8 × 10 ^–19^	4.5 × 10^–18^
2	hsa05220	Chronic myeloid leukemia	12	1.3 × 10 ^–15^	1.6 × 10^–14^
3	hsa05219	Bladder cancer	9	1.3 × 10 ^–13^	1.1 × 10^–12^
4	hsa05213	Endometrial cancer	9	1.3 × 10 ^–12^	8.4 × 10^–12^
5	hsa05223	Non-small cell lung cancer	8	8.2 × 10^–11^	4.1 × 10^–10^
6	hsa05210	Colorectal cancer	9	2.0 × 10^–10^	8.4 × 10^–10^
7	hsa05214	Glioma	8	4.6 × 10^–10^	1.7 × 10^–9^
8	hsa05216	Thyroid cancer	6	8.1 × 10^–10^	2.5 × 10^–9^
9	hsa05218	Melanoma	8	1.0 × 10^–9^	2.9 × 10^–9^
10	hsa05212	Pancreatic cancer	8	1.2 × 10^–9^	3.0 × 10^–9^
11	hsa05221	Acute myeloid leukemia	7	4.4 × 10^–9^	1.0 × 10^–8^
12	hsa04012	ErbB signaling pathway	8	6.7 × 10^–9^	1.4 × 10^–8^
13	hsa05211	Renal cell carcinoma	7	2.1 × 10^–8^	4.0 × 10^–8^
14	hsa04110	Cell cycle	8	1.1 × 10^–7^	1.9 × 10^–7^
15	hsa05222	Small cell lung cancer	7	1.2 × 10^–7^	2.0 × 10^–7^
16	hsa04630	Jak-STAT signaling pathway	8	1.0 × 10^–6^	1.6 × 10^–6^
17	hsa04520	Adherens junction	6	1.1 × 10^–6^	1.6 × 10^–6^
18	hsa04916	Melanogenesis	6	6.6 × 10^–6^	9.1 × 10^–6^
19	hsa04310	Wnt signaling pathway	7	9.6 × 10^–6^	1.3 × 10^–5^
20	hsa04660	T cell receptor signaling pathway	5	4.9 × 10^–5^	6.2 × 10^–5^
21	hsa04510	Focal adhesion	7	7.8 × 10^–5^	9.3 × 10^–5^
22	hsa04650	Natural killer cell mediated cytotoxicity	5	3.7 × 10^–4^	4.2 × 10^–4^
23	hsa04120	Ubiquitin mediated proteolysis	5	4.0 × 10^–4^	4.3 × 10^–4^
24	hsa04010	MAPK signaling pathway	7	5.6 × 10^–4^	5.9 × 10^–4^

^a^Number of genes in the 44 cancer protein set that could be found in KEGG pathways.

^b^*P* values were calculated by the Fisher’s exact test.

^c^FDR *P* values were calculated using Benjamini-Hochberg procedure.

## Conclusion

In this study, we explored the global characteristics of cancer proteins in the human protein- protein interaction network. Based on the four topological measurements, we found that the cancer proteins had significantly different topological properties compared to the essential or control proteins. Specifically, cancer proteins tended to have higher connectivity and betweenness, shorter shortest-path distance, and weaker clustering coefficient than other proteins. We also found recessive cancer proteins had even stronger network characteristics than dominant cancer proteins. Our results suggest that protein-protein interaction features of cancer genes are important for our understanding the etiology of cancers and, potentially, for other complex diseases.

## Competing interests

The authors declare that they have no competing interests.

## Authors’ contributions

JS participated in the method development, prepared the data, carried out the data analysis, and contributed to the writing of the manuscript. ZZ participated in the method development and data analysis, and contributed to the writing of the manuscript. All authors read and approved the final manuscript.
